# Self-referral practice among patients attending outpatient department of Northwest Comprehensive Specialized Hospital, Ethiopia: a multi-center cross-sectional study

**DOI:** 10.3389/fpubh.2025.1434669

**Published:** 2025-05-23

**Authors:** Tihtna Alemu, Zeamanuel Anteneh Yigzaw, Mekides Engeda Alemu, Lakew Asmare

**Affiliations:** ^1^Department of Surgical Nursing, School of Nursing, College of Medicine and Health Sciences, University of Gondar, Gondar, Ethiopia; ^2^Department of Health Promotion and Behavioral Science, School of Public Health, College of Medicine and Health Sciences, Bahir Dar University, Bahir Dar, Ethiopia; ^3^Department of Surgical Nursing, School of Nursing and Midwifery, College of Medicine and Health Sciences, Debre Berhan University, Debre Berhan, Ethiopia; ^4^Department of Epidemiology and Biostatistics, Institute of Public Health, College of Medicine and Health Sciences, University of Gondar, Gondar, Ethiopia

**Keywords:** associated factors, Northwest Ethiopia, outpatient department, referral system, self-referral practice

## Abstract

**Background:**

In resource-scarce countries such as Ethiopia, patients coming from primary care facilities directly to specialist hospitals pose an enormous challenge to the health system. This practice hinders continuity of care and reduces the quality of healthcare. However, little is known about the prevalence of self-referral in Ethiopia. Therefore, this study aimed to assess the self-referral practice and its associated factors in Northwest Comprehensive Specialized Hospital, Ethiopia.

**Methods:**

A multi-center institutional-based cross-sectional design was conducted. A systematic random sampling technique was used to recruit a total of 446 participants. Structured interviewer-administered questionnaires were used. The collected data were entered into EpiData software and exported for analysis. Variables with a *p*-value of <0.25 in the bi-variable analysis were considered candidates for multivariable analysis. A *p*-value of <0.05 and a 95% confidence interval were used to determine the statistical association.

**Results:**

The prevalence of self-referral practice was 339 (70.40%). Thus, respondents who had an education level of college and above [AOR = 0.35, 95% CI = 0.10, 0.92], respondents who perceived their illness [AOR = 1.11, 95% CI = 1.06, 2.53], and those who had adequate knowledge about the referral system (AOR = 0.31, 95%CI = 0.16, 0.68) were significantly associated variables with self-referral practice.

**Conclusion:**

According to this study’s findings, more patients than expected visited specialized hospitals than previously reported. Various factors, such as educational level, perceived severity of illness, confidence in laboratory services, lack of information about referral methods, and limited use of primary care, contribute to this issue. Addressing these factors and strengthening the referral process are important steps to improve healthcare delivery, continuity of care, and patient satisfaction in Ethiopia.

## Background

Self-referral is a phenomenon in which patients bypass one of the primary healthcare facilities (PHCFs) and go directly to a higher-level care facility (1). This results in the underutilization of primary care facilities, overburdening and congestion of referral facilities, increased healthcare costs, increased waiting time of patients, disruption of continuity of care, decreased quality of the healthcare system, and decreased patient satisfaction (2–8). Globally, the prevalence of self-referral showed that approximately 44–60% of patients seek care at a higher-level health facility instead of visiting a PHCF. Studies conducted in a different African country showed that the prevalence of bypassing primary healthcare facilities without regard accounted for 35–87% (9–13). Similarly, studies conducted in Ethiopia showed that the prevalence of self-referral in general and referral hospitals was 82 and 84.4%, respectively (2). Other evidence has shown that improving the availability of medication and diagnostic facilities, improving the quality of PHC services, and increasing patients’ awareness of the referral system are solutions to improve the practice of self-referral (2, 4, 5).

Ethiopia’s new healthcare plan prioritizes a three-tier system: primary (local facilities), secondary (general hospitals), and advanced (specialty hospitals). Strengthening connections and communication across these levels is crucial for the plan to be successful (14, 15). However, healthcare utilization at PHCFs is still low when compared with the accelerated expansion of primary healthcare facilities (16). The Federal Ministry of Health (FMoH) guidelines for implementing a patient referral system state that, except in emergency cases, all patients should first be seen at PHCFs, and if a referral is necessary, healthcare providers refer to referral facilities, which also send feedback (17). However, previous studies assessed self-referral at general hospitals and single settings (2, 18); self-referral was not assessed at specialized hospitals with a multi-center setting in our country. Therefore, this study aimed to assess self-referral practice-associated factors among patients at adult outpatient departments at Northwest Comprehensive Specialized Hospital, Ethiopia.

## Methods

### Study design and period

A multi-center institution-based cross-sectional study was conducted at the Northwest Comprehensive Specialized Hospital between 20 April 2020 and 25 May 2020.

### Study area

According to the Amhara National Regional Health Bureau’s Annual Performance Report, the region has 81 hospitals, 858 health centers, and 3,560 health posts (19). Of the 81 hospitals, 5 are comprehensive specialized hospitals located in Northwest Ethiopia: the University of Gondar Comprehensive Specialized Hospital (UoGCSH), Tibebe Gion Comprehensive Specialized Hospital (TGCSH), Debre Tabor Comprehensive Specialized Hospital (DTCSH), Felege Hiwot Comprehensive Specialized Hospital (FHCSH), and Debre Markos Comprehensive Specialized Hospital (DMCSH). Based on the reports from the outpatient department coordinators of the five hospitals, approximately 29,700 outpatients were served.

### Population

All patients attending the outpatient department of a comprehensive specialized hospital in Northwest Ethiopia were the source population. All patients aged >18 years who visited the outpatient department during the study period were in the study population. However, patients with emergency cases were excluded from the study.

### Sample size and sampling procedure

The sample size was determined using a single population proportion formula, assuming *p* = 82.00% from a previous similar study conducted in the country (2), with a margin of error (d) of 0.05 and a 95% confidence interval. Therefore, the final sample size with a 10% non-response rate was 249. A double proportion formula was used for the second objective, considering significant variables such as knowledge of the referral system, perception of illness, and availability of laboratory services. The largest calculated sample size was selected, resulting in a final sample size of 447 participants.

A systematic random sampling technique was employed. This involved considering the average number of outpatient visits to the hospital per month and determining the sampling interval (k) to select respondents accordingly. Based on the hospital’s outpatient department head report, an average of 300 patients visited the UoGCSH and TGCSH per day, and 250 patients visited DTCSH, FHCSH, and DMCSH per day. Then, samples from each specialized hospital were proportionally allocated, which was estimated based on their average visit to the specialized hospitals. Following the sample’s proportionate distribution to each specialized hospital, 447 samples (UoGCSH = 99, TGCSH = 97, DTCSH = 86, FHCSH = 80, and DMCSH = 84) were used. This led to taking approximately every fifth interval from each hospital and selecting the first participant using the lottery method.

### Measurements of variables

Self-referral practice: This was measured if the respondents answered “No” to the question “Were you referred from a primary health facility to this comprehensive specialized hospital.” It was coded “1” if respondents answered “No” and “0” if respondents answered “Yes” (2).

Patient knowledge of the referral system: This was measured by five “Yes/No” questions. If the study participants answered correctly above the mean (mean = 2.14), he/she was considered to have adequate knowledge; otherwise, he/she was considered to have inadequate knowledge (17).

### Data collection tools and procedures

Data were collected using a structured and pre-tested interviewer-administered questionnaire adapted from different literature (2, 7–9, 11). First, the questionnaires were prepared in English, then translated into the local language, Amharic, and translated back to English to check consistency by an independent translator. A total of 18 questionnaires were administered, which consisted of sociodemographic characteristics (consisting of 5 questions) and institution-related questions (consisting of 13 questions). The principal investigator provided training for data collectors about the data collection process. The data were collected by two trained Bachelor of Science (BSc) Nurses and supervised by one Master of Science (MSc) nurse after training was provided for both data collectors and supervisors.

### Data quality control

The Amharic version of the questionnaires was used to collect the data. A pre-test was performed by the principal investigator at Dessie Comprehensive Specialized Hospital among 22 study participants before the study period, and logistical issues in the flow of the questionnaire were identified by modifying the data collection tool. Supervisors and data collectors received 2 days (approximately 12 h) of training covering the goal, the purpose, patient approach, confidentiality, and consent-taking techniques. The principal investigator and the lead investigator closely monitored the process and made due corrections. Before entering the data, the questionnaire’s completeness was verified and manually cleaned.

### Data processing and analysis

The data were exported from EpiData version 3.01 to STATA version 14 for analysis. Descriptive and summary statistics were presented in the form of text and tables. The chi-square (χ^2^) assumption was checked for all independent variables, and all variables passed. Multicollinearity among independent variables was checked using the variance inflation factor (VIF) and was found to be less than 10 (mean value = 1.5), which was acceptable (20). To determine the potential variables for multivariable analyses and to examine the connection between the independent and dependent variables, a binary logistic regression analysis was used. To account for confounding variables, independent variables from the bivariable logistic regression analyses with a *p*-value of <0.25 were included in the multivariable logistic regression analysis. Finally, factors related to self-referral were identified using an adjusted odds ratio (AOR) with a 95% confidence interval and a *p*-value of <0.05. Model fitness was tested using the Hosmer–Lemeshow goodness-of-fit test (*p* = 1.04).

## Results

### Sociodemographic characteristics

A total of 447 study participants, with a response rate of 99.8%, participated in the study. The mean age of the respondents was 34.5 with ± 14.2 standard deviation. The majority (33%) of respondents were in the age group of 25–34 years (Table 1).

**Table 1:** Sociodemographic characteristics of respondents at the Northwest Comprehensive Specialized Hospital, 2020 (*N* = 446).

Variables	Categories	Frequency	Percent (%)
Age in years	17–24	120	26.9
	25–34	147	33.0
	35–44	76	17.0
	>45	103	23.1
Sex	Male	183	41.0
	Female	263	59.0
Marital status	Married	243	54.49
	unmarried	203	45.51
Educational status	Unable to write and read	124	27.8
	Able to write and read	59	13.2
	Primary school	76	17.0
	Secondary school	75	16.8
	College and above	112	25.1
Occupation	Government	58	13.0
	Merchant	84	18.8
	Farmer	92	20.6
	Housewife	133	29.8
	Self-employee	73	16.4
	Others	6	1.3

Unmarried = single, divorced, and separated; others = students and unemployed.

### Institutional factors

In this study, the prevalence of practiced self-referral was 339 (70.40%) with 95% CI = 68.10, 76.30%. Approximately 240 (70.8%) respondents practiced self-referral due to a lack of laboratory services in PHCFs, and approximately 78 (23%) respondents were due to the closeness of the specialized hospital to their place of residence (Table 2).

**Table 2:** Institutional factors of self-referral practice among patients attending the outpatient department at the Northwest Comprehensive Specialized Hospital, 2020 (*n* = 446).

Variables	Categories	Frequency	Percent (%)
Referred from PHCFs	Yes	107	29.6
	No	339	70.4
Availability of drugs at PHCFs	Yes	118	34.8
	No	221	65.2
Availability of the laboratory at PHCFs	Yes	99	29.2
	No	240	70.8
Distance from the hospital	>5 km	277	81.7
	≤5 km	62	18.3
Availability of transportation	No	276	81.4
	Yes	63	18.6
Nearby health facility	Health center	261	77.0
	Specialized Hospital	78	23.0
Waiting time at PHCFs	>30 min	166	37.2
	≤30 min	280	62.8
Previous experience with PHCFs	No	203	45.5
	Yes	243	54.5
Knowledge	Adequate	195	43.7
	Inadequate	251	56.3

### Factors associated with practiced self-referral

In this model, the following variables were included in the multivariable regression because their overall *p*-value in the bi-variable regression was *<*0.25. Thus, the odds of self-referral practice decreased by 55% [AOR = 0.45, 95% CI = 0.10, 0.62] among respondents with an education level of college and above compared to those whose educational level was unable to read and write. The odds of self-referral practice decreased by 69% (AOR = 0.31, 95%CI = 0.16, 0.68) among respondents who had adequate knowledge about the referral system compared to those who had inadequate knowledge about the referral system. Moreover, the odds of self-referral practice were increased by 1.11 times [AOR = 1.11, 95% CI = 1.06, 2.53] among respondents who perceived their illness as severe compared to their counterparts (Table 3).

**Table 3:** Bivariable and multivariable analyses of self-referral practice among patients attending the outpatient department at Northwest Comprehensive Specialized Hospital, 2020 (*n* = 446).

Variables	Categories	Self-referral practice		COR [95% CI]	AOR [95% CI]
		Yes	No		
Education	Cannot read and write	63	57	1.00	1.00
	Formal education	10	43	0.41 [0.07, 3.23]	0.36 [0.60, 3.58]
	1–8 grade	20	57	0.62 [0.09, 4.63]	0.31 [0.08, 3.08]
	9–12 grade	102	107	0.40 [0.04, 0.95]*	0.35 [0.10, 10.19]
	College and above	89	96	0.83 [0.30, 1.01]**	0.45 [0.10, 0.62]*
Information for referral	No	106	169	1.00	1.00
	Yes	71	100	1.13 [1.03, 3.54]**	0.92 [0.52, 1.63]
Recommended by others to visit PHF	No	88	127	1.00	1.00
	Yes	95	107	0.78[0.52, 1.14]	0.41 [0.19, 1.94]
Confident in the availability of the laboratory	No	23	228	0.13 [0.10,0.82] **	0.11 [0.08,1.75]
	Yes	84	111	1.00	1.00
Knowledge	Inadequate	128	192	1.00	1.00
	Adequate	79	147	0.80 [0.22, 0.99] **	0.31 [0.16, 0.68] *
Perceived severity of illness	Mild	25	59	1.00	1.00
	Moderate	36	206	0.41 [0.44, 5.60]	0.33 [0.41, 2.08]
	Severe	46	74	1.46 [1.07, 3.66]**	1.11 [1.06, 2.53]*

**p < 0.25, *p-value <0.05, COR, crude odds ratio; AOR, adjusted odds ratio; CI, confidence interval, and 1.00, reference.

## Discussion

The study showed that the prevalence of self-referral practice was 70.40% with 95% CI = 68.10, 76.30%. This finding was in line with other studies conducted in Iran (75%) (21) and Klaipeda City, Lithuania (69%) (18). This finding was also higher than other studies conducted in Japan (14%) (9), China (9–66%) (6), Swaziland (60%) (21), Nigeria (39.7%) (7), South Africa (36%) (8), and Tanzania (59%) (4). However, this finding was lower than other studies conducted in Sudan (87%) (19) and Nekemte, Ethiopia (82%) (2). This disparity may be due to different technological advancements, healthcare infrastructure, referral enforcement, cultural norms in healthcare-seeking, and sociodemographic characteristics of the population in the setting (22). For instance, countries with well-established and strictly enforced referral systems, such as Japan, tend to have lower self-referral rates (14). In contrast, in settings where access to primary care is limited or perceived as inadequate, patients may be more inclined to bypass lower-level facilities.

The findings showed that those with a college-level education or higher were less likely to engage in self-referral practices. A possible explanation is that higher education levels may increase the knowledge level related to self-referral practice in different aspects and highlight the importance of public awareness and education campaigns to promote adherence to the referral system. Moreover, respondents with adequate knowledge about the referral system were less likely to practice self-referral. This finding is supported by other studies conducted in Nekemte, Ethiopia (2), and South Africa (8). This may be because respondents who are aware of the referral system tend to ask for a referral letter before coming to the referral hospital, and they primarily visit the PHCFs before coming to specialized hospitals.

Conversely, respondents who perceived their illness as severe were more likely to practice self-referral than those who perceived their illness as mild. This finding is supported by studies conducted in Nekemte, Ethiopia (2), China, and Niger (5, 23). This may be due to a lack of confidence in the diagnostic and treatment capabilities of PHCFs or a perception that seeking care directly at a specialized hospital will expedite their treatment (16, 24).

The high rate of self-referral observed in this study has significant implications for healthcare delivery and resource allocation in Ethiopia. The over-utilization of specialized hospitals for conditions that could be managed at the primary care level contributes to congestion, increased waiting times, and potentially higher healthcare costs (2). Furthermore, it undermines the intended function of the three-tier healthcare system, which aims to provide accessible and continuous care at the primary level (17). Implementing public awareness campaigns is crucial to educate the population about the benefits of utilizing PHCFs for non-emergency conditions, adhering to the referral system, enhancing the referral system through clear guidelines and improved communication, addressing perceptions of illness severity via provider training, and bolstering community health programs can collectively reduce self-referral, optimize healthcare resource allocation, and improve patient outcomes, aligning with the Ethiopian Federal Ministry of Health’s strategic goals for a three-tiered healthcare system (17, 25).

## Strengths and limitations of the study

This study assessed different factors for self-referral practice, which provides updated information. Moreover, it may serve as the basis for future studies, and the findings can inform healthcare policies and interventions. As a limitation, the study relied on self-reported data, which is susceptible to recall bias. Respondents may not recall or accurately report their prior behaviors in seeking healthcare or why they decided to self-refer to the hospital. Additionally, this study does not assess broader health-seeking behavior.

## Conclusion

The study showed that the prevalence of self-referral practices was found to be high. Various factors such as educational level, perceived severity of illness, availability of laboratory services, knowledge about the referral system, and use of primary care contributed to this issue. Based on these findings, policymakers should consider local factors/contexts when making decisions about strengthening the referral system in Ethiopia.

## Data Availability

The original contributions presented in the study are included in the article. Further inquiries can be directed to the corresponding author/s.
